# Diagnostic Accuracy of Tuberculosis Screening Tests in a Prospective Multinational Cohort: Chest Radiography With Computer-Aided Detection, Xpert Tuberculosis Host Response, and C-Reactive Protein

**DOI:** 10.1093/cid/ciae549

**Published:** 2024-11-07

**Authors:** Rebecca Crowder, Balamugesh Thangakunam, Alfred Andama, Devasahayam J Christopher, Victoria Dalay, Welile Nwamba, Sandra V Kik, Dong Van Nguyen, Nguyen Viet Nhung, Patrick P J Phillips, Morten Ruhwald, Grant Theron, William Worodria, Charles Yu, Payam Nahid, Adithya Cattamanchi, Ankur Gupta-Wright, Claudia M Denkinger, for the R2D2 TB Network, Shanmugasundaram Elango, Jerusha Emmanuel, Vinita Ernest, Priyadarshini Gajendran, Flavita John, Bharath Karthikeyan, Divya Mangal, Swetha Sankar, Rajasekar Sekar, Reena Sekar, Deepa Shankar, Mary Shibiya, Sai Vijayasree, Jared Almonte, Kevin Joshua Alonzo, Mary Faith Angcaya, Joseph Edwin L Bascuña, Ramon P Basilio, Asella Ruvijean Cariaga, Gabriella Castillon, Karlo Dayawon, Raul Destura, Jezreel Esguerra, Eleonor Garcia, Darecil Gelina, Joseph Aldwin Goleña, Maria Marissa Golla, Emmanuelle Gutierrez, Gidalthi Jonathan Ilagan, Dodge R Lim, Jaiem Maranan, Danaida Marcelo, Leonedy Masangcay, Jenkin Mendoza, Angelita Pabruada, Laarean Perlas, Annalyn Reyes, Roeus Vincent Arjay G Reyes, Lorenzo Reyes, Maria Guileane Sanchez-Pogosa, Maricef Tonquin, Shima Abdulgadar, Cammy Botha, Brigitta Derendinger, Jane Fortuin, Siphosethu Gonya, Chumani Hatile, Megan Hendrikse, Charlotte Lawn, Disha Mathoorah, Desiree Lem Mbu, Zintle Ntetha, Anna Okunola, Zaida Palmer, Fikiswa Seti, Charmaine Van Der Walt, Lusanda Yekani, Lucy Asege, Alice Bukirwa, David Katumba, Esther Kisakye, Wilson Mangeni, Job Mukwatamundu, Sandra Mwebe, Annet Nakaweesa, Martha Nakaye, Talemwa Nalugwa, Irene Nassuna, Irene Nekesa, Justine Nyawere, John Baptist Ssonko, Hai Dang, Luong Dinh, Hang Do, Tam Do, Thuong Do, Dung Dao, Ha Doan, Thien Doan, Huy Ha, Oanh Lai, Hien Le, Nguyet Le, Anh Nguyen, Hanh Nguyen, Hoa Nguyen, Hoang Nguyen, Thanh Nguyen, Yen Nguyen, Ha Phan, Nam Pham, Thuong Pham, Trang Trinh, Phuong Vu, Trung Vu, Robert Castro, Adithya Cattamanchi, Catherine Cook, Sophie Huddart, Devan Jaganath, Midori Kato-Maeda, Tessa Mochizuki, Ruvandhi Nathavitharana, Payam Nahid, Kevin Nolan, Kinari Shah, Swati Sudarsan, Christina Yoon, Maria del Mar Castro Noriega, Theresa Pfurtscheller, Seda Yerlikaya, Matthew Arentz, Nathalie Frey

**Affiliations:** Center for Tuberculosis and Division of Pulmonary and Critical Care Medicine, University of California, San Francisco, San Francisco, California, USA; Department of Pulmonary Medicine, Christian Medical College, Vellore, India; Department of Medicine, Makerere University College of Health Sciences, Kampala, Uganda; Department of Pulmonary Medicine, Christian Medical College, Vellore, India; Department of Microbiology and Parasitology, College of Medicine, De la Salle Medical and Health Sciences Institute, Dasmariñas, Philippines; DSI-NRF Centre of Excellence for Biomedical Tuberculosis Research, South African Medical Research Council Centre for Tuberculosis Research, Division of Molecular Biology and Human Genetics, Faculty of Medicine and Health Sciences, Stellenbosch University, Cape Town, South Africa; TB Programme, FIND, Geneva, Switzerland; Network Management Department, Ha Noi Lung Hospital, Ha Noi, Vietnam; VNU University of Medicine and Pharmacy, National Lung Hospital, Ha Noi, Vietnam; Center for Tuberculosis and Division of Pulmonary and Critical Care Medicine, University of California, San Francisco, San Francisco, California, USA; TB Programme, FIND, Geneva, Switzerland; DSI-NRF Centre of Excellence for Biomedical Tuberculosis Research, South African Medical Research Council Centre for Tuberculosis Research, Division of Molecular Biology and Human Genetics, Faculty of Medicine and Health Sciences, Stellenbosch University, Cape Town, South Africa; Department of Medicine, Makerere University College of Health Sciences, Kampala, Uganda; Department of Internal Medicine, College of Medicine, De La Salle Medical and Health Sciences Institute, Dasmariñas, Philippines; Center for Tuberculosis and Division of Pulmonary and Critical Care Medicine, University of California, San Francisco, San Francisco, California, USA; Division of Pulmonary Diseases and Critical Care Medicine, University of California Irvine, Irvine, California, USA; Department of Infectious Disease and Tropical Medicine, Heidelberg University Hospital, German Center for Infection Research (partner site), Heidelberg, Germany; Department of Infectious Diseases, Imperial College London, London, United Kingdom; Department of Infectious Disease and Tropical Medicine, Heidelberg University Hospital, German Center for Infection Research (partner site), Heidelberg, Germany

**Keywords:** Tuberculosis, diagnostics, screening, triage

## Abstract

**Background:**

Accessible, accurate screening tests are necessary to advance tuberculosis case finding and early detection in high-burden countries.

**Methods:**

We prospectively screened adults with ≥2 weeks of cough at primary health centers in the Philippines, Vietnam, South Africa, Uganda, and India. Participants underwent chest radiography, Cepheid Xpert TB Host Response (Xpert HR) testing, and point-of-care C-reactive protein (CRP) testing (Boditech). Chest radiographs were processed using CAD4TB v7, a computer-aided detection (CAD) algorithm. We assessed diagnostic accuracy against a microbiologic reference standard (sputum Xpert Ultra, culture). Optimal cutoff points were chosen to maximize specificity at 90% sensitivity. Two-test screening algorithms were considered, using (1) sequential negative serial screening (with positive defined as positive on either test) and (2) sequential positive serial screening (with positive defined as positive on both tests).

**Results:**

Between July 2021 and August 2022, a total of 1392 participants with presumptive tuberculosis had valid index tests and reference standard results, and 303 (22%) had confirmed tuberculosis. In head-to-head comparisons, CAD4TB v7 showed the highest specificity at 90% sensitivity (70.3% vs 65.1% for Xpert HR [95% confidence interval for absolute difference in specificity, 1.6%–8.9%] and vs 49.7% for CRP [17.0%–24.3%]). Three 2-test screening algorithms met World Health Organization target product profile minimum accuracy thresholds and had higher accuracy than any test alone. At 90% sensitivity, the specificity was 79.6% for Xpert HR–CAD4TB (sequential negative), 75.9% for CRP-CAD4TB (sequential negative), and 73.7% for Xpert HR–CAD4TB (sequential positive).

**Conclusions:**

CAD4TB achieves target product profile targets and outperforms Xpert HR and CRP. Combining screening tests further increased accuracy.

**Clinical Trials Registration.** NCT04923958

## Teaser

Our study compares the diagnostic accuracy of 3 available tuberculosis screening tests (chest radiograph processed with CAD4TB, Xpert TB Host Response, and C-reactive protein) among people with presumptive tuberculosis in 5 countries (Uganda, South Africa, Philippines, Vietnam, and India).

Approximately 7.5 million people had tuberculosis diagnosed in 2022, and 1.3 million died from the disease [1]. Globally, 3.1 million tuberculosis cases were undiagnosed or unreported in 2022 [1]. This demonstrates that access to high-quality and affordable tuberculosis testing remains limited, making tuberculosis diagnosis the weakest link in the tuberculosis cascade of care [2]. World Health Organization (WHO) target product profiles (TPPs) for tuberculosis diagnostics prioritize a simple, low-cost, non–sputum-based point-of-care triage or screening test to rule out tuberculosis and guide confirmatory testing [3].

Promising tuberculosis screening tests include improvements on established technologies, such as chest radiography [4]; new application of existing tests, such as C-reactive protein (CRP); and novel assays, including the Xpert TB Host Response (Xpert HR) cartridge (Cepheid). Implementation of chest radiography for tuberculosis screening has been limited in resource-constrained settings due to centralized chest radiographic infrastructure [5] and scarcity of skilled personnel for interpretation [6]. The advent of digital chest radiography with computer-aided detection (CAD) analysis may reduce barriers to chest radiographic screening by quickly enabling screening without the need for a skilled reader [7, 8]. However, performance varies by population screened, and limited data are available for children [9–12].

CRP is a nonspecific marker for inflammation that has shown better accuracy than symptom screening for tuberculosis among people with human immunodeficiency virus (HIV) [13]. High CRP levels are correlated with mycobacterial load and associated with poor prognostic clinical features and a higher risk of death [14, 15]; however, CRP alone has not been shown to meet TPP specificity targets [13, 14, 16]. The recently developed Xpert HR assay detects expression levels of host genes in whole blood. Results from early studies found that this assay approaches WHO minimum accuracy targets for a non–sputum-based triage test [17, 18].

Direct comparison of diagnostic accuracy, alongside comparisons of cost and operational characteristics, are needed to guide policy decisions around the implementation of novel tuberculosis screening tests and assess consistency with TPP targets. Testing algorithms that combine available tests may be needed to meet desired characteristics. Chest radiography with CAD reading is considered a possible second screening test by WHO, which could be used in conjunction with a simpler point-of-care screening test (e.g., CRP). We aimed to conduct a head-to-head comparison of the diagnostic accuracy of 3 potential tuberculosis screening tests, including CAD, CRP, and Xpert HR in a large multi-country cohort of people with presumptive tuberculosis.

## METHODS

### Study Design, Population, and Procedures

In this prospective study, we screened adults (≥18 years) presenting to primary health centers with presumed tuberculosis between July 2021 and August 2022 in the Philippines, Vietnam, Uganda, South Africa, and India. Specific enrollment locations have been reported elsewhere [18] and are listed in the Supplementary Materials (Supplementary Table 1). We enrolled consecutive outpatients if they had ≥2 weeks of new or worsening cough. We excluded people who had completed treatment for tuberculosis infection or disease within the past 12 months, had taken antibiotics with antimycobacterial activity within 2 weeks of study entry, or were unable or unwilling to return for follow-up or provide informed consent. All participants underwent HIV and diabetes screening (using hemoglobin A1c [HbA1c]), and 2–3 spot sputum samples were collected for reference standard testing (sputum was induced if the participant was unable to expectorate spontaneously). Study participants underwent chest radiography, provided venous or capillary blood for Xpert HR testing, and provided capillary blood for CRP testing.

### Index Testing

All participants underwent antero-posterior or postero-anterior chest radiography at baseline. If digital chest radiography was not available, analogue chest radiographic images were scanned to create jpeg images, which were then converted into DICOM format using the img2dcm tool from the dcmtk toolkit (version 3.6.6). Images that did not fulfill the DICOM features required for successful CAD software processing were subsequently modified using the dcmodify tool (version 3.6.6) from the dcmtk toolkit before they were processed with CAD software. Images were processed using 5 different CAD algorithms, but based on previous analysis [19], we chose the best-performing CAD algorithm, CAD4TB v7 (CAD4TB; Delft Imaging), to be evaluated for comparisons in this analysis. CAD analysis was conducted by the Foundation for Innovative New Diagnostics (FIND), according to the developers’ instructions. CAD4TB produces a tuberculosis score, ranging from 0 to 100.

The Xpert MTB-HR research-use-only prototype cartridge (Cepheid) evaluates messenger RNA levels of 4 differentially expressed genes (*GBP5, DUSP3, KLF2,* and *TBP*). Testing involved inoculating the cartridge by transferring 100 µL of freshly collected whole or capillary blood in an ethylenediaminetetraacetic acid tube into the cartridge chamber and analyzing using the GeneXpert instrument as per manufacturer instructions. We calculated the “tuberculosis score” using the formula (Ct *GBP5* + Ct *DUSP3*)/2 – Ct *TBP*, as described elsewhere [18], where Ct represents cycle threshold.

CRP concentrations were measured at baseline from capillary blood using a US Food and Drug Administration–approved standard sensitivity point-of-care assay, measured using ichroma II (Boditech), which provides quantitative results in 3 minutes (range, 2.5–300 mg/L). A fixed-volume micropipette (50 µL) was used to obtain and transfer capillary blood to a reagent tube, the tube was inverted 10 times, two drop were discarded, and then 2 drops were applied on the lateral-flow device for testing. No prespecified cutoff point was provided for any index test. All operators performing the index and reference tests were blinded to clinical details and other tuberculosis testing results.

### Reference Standard Testing

Each participant provided 2–3 sputum samples, and the microbiologic reference standard (MRS) was determined by results from sputum Xpert Ultra MTB/RIF (Cepheid) and mycobacterial culture (MGIT; Becton Dickinson; solid culture in 7H10 medium was used when MGIT supplies were not available). A positive reference standard was defined as a positive sputum Xpert Ultra result (grade “very low” or higher or 2 “trace” results) or a positive culture. Participants with a positive Xpert Ultra result did not undergo confirmatory culture. A negative reference standard was defined as no positive results with Xpert Ultra or culture and 2 negative cultures. Those not meeting the criteria for positive or negative were defined as indeterminate and excluded from the analysis. We also used a secondary, simplified sputum Xpert Ultra reference standard, given that a molecular test is typically the only confirmatory tuberculosis test available in programmatic settings.

### Statistical Analysis

For each index test, we conducted receiver operating characteristic (ROC) analysis to compute the area under the curve (AUC) with 95% confidence interval (CI). Participants with indeterminate index test results were excluded from the analysis. For the primary analysis, optimal cutoff points were chosen for each test that would achieve the TPP target sensitivity ≥90% and maximize specificity in the entire study population. Two-test screening algorithms were also considered. We considered (1) a sequential negative serial screening approach [20], in which the second screening test is conducted only if the first result is negative and a positive screen is defined as positive on either test, and (2) a sequential positive serial screening approach [20], in which the second screening test is conducted only if the first is positive and a positive screen is defined as positive on both tests (Supplementary Figure 1).

For each potential combination, we considered 1000 possible combinations of cutoff points (100 possible cutoffs for the first and 100 possible cutoffs for the second test). We then identified all pairs of cutoffs that would achieve sensitivity ≥90% and specificity ≥70%, then likewise selected the pair that achieved the highest possible specificity within these constraints. With all binary tests set to have a sensitivity of 90%, our primary analysis compared the specificity of each index alone and the possible combined 2-test screening algorithms among the entire study population. The absolute difference in specificity for each pairwise comparison was calculated using McNemar test for paired proportions. Results are reported according to STARD guidance [21]. Secondary analyses compared the sensitivity and specificity of the index tests alone and in combination among subgroups of interest (by country, HIV status, diabetes status). A sample size of 1500 (300 per country) was prespecified to achieve reasonable CI widths between 5.2% and 9.4%, depending on observed test sensitivity (80%–95%), and 4.6%–5.6%, depending on observed test specificity (60%–80%), assuming a tuberculosis prevalence of 20%. Stata software, version 18, was used for all analyses.

### Ethics

This study was registered with ClinicalTrials.gov (NCT04923958). Study procedures were approved by the institutional review board of the University of California, San Francisco, the University of Heidelberg, and local institutional review boards at each enrollment site (Supplementary Table 1).

## RESULTS

### Participants

**Figure 1.**
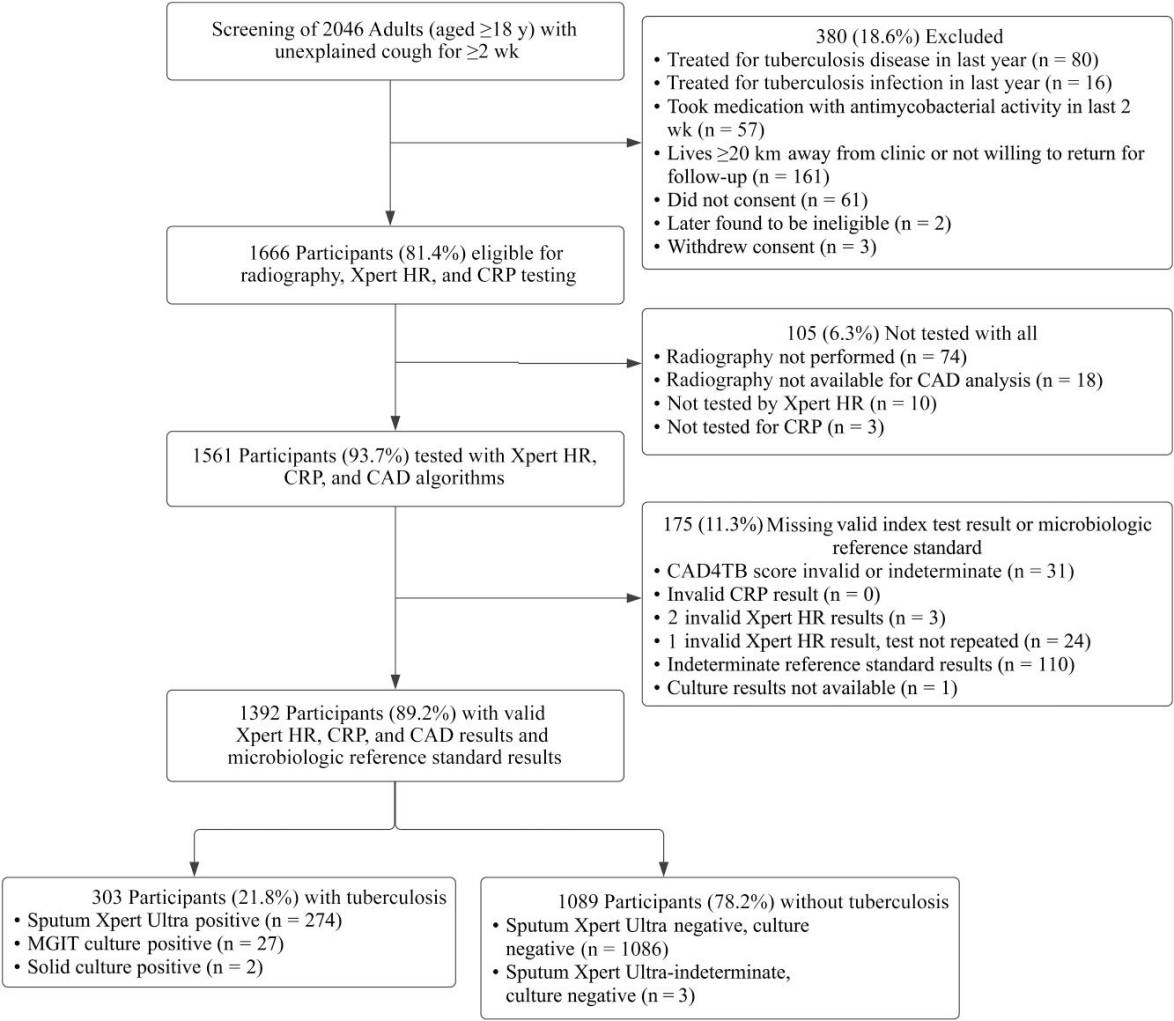
Study population. Note that culture was not performed for participants with positive sputum Xpert Ultra results. Abbreviations: CAD, computer-aided detection; CRP, C-reactive protein; MGIT, mycobacterial culture; Xpert HR, Cepheid Xpert TB Host Response.

Between July 2021 and August 2022, 2046 adults were screened across clinical sites in 5 countries (the Philippines, Vietnam, Uganda, South Africa, and India). Of these, 1666 (81%) met the criteria for inclusion in the study, of whom 1561 (94%) underwent all 3 tests; 31 (2%) of the CAD4TB readings, 27 (2%) of the Xpert HR results, and no CRP results were indeterminate or invalid; 1392 participants (89%) had all 3 index tests and the MRS performed with valid results (Figure 1). Of the 1392 assessable participants, 46% were female, the median age was 43 years, 14% were diagnosed with HIV (median CD4 cell count, 378/µL), and 14% were diagnosed with diabetes (median HbA1c, 7.0) (Table 1). In total, 303 (22%) had microbiologically confirmed tuberculosis. However, characteristics varied by country (Table 1), with tuberculosis prevalence ranging from 9% in India and the Philippines to 38% in Vietnam.

**Table 1.** Demographic and Clinical Characteristics by Country

Characteristic	Study Participants, No. (%)^a^					
	Total (n = 1392)	Philippines (n = 326)	Vietnam (n = 276)	Uganda (n = 267)	South Africa (n = 262)	India (n = 261)
Age, mean (SD), y	43 (16)	40 (15)	51 (16)	34 (12)	40 (12)	48 (16)
Female sex	633 (45)	181 (56)	108 (39)	112 (42)	125 (48)	107 (41)
BMI, mean (SD)^b^	23 (5)	24 (5)	20 (3)	22 (4)	24 (7)	23 (6)
PWH^c^	197 (14)	3 (1)	2 (1)	80 (30)	102 (40)	10 (4)
CD4 cell count, median (IQR), cells/µL	378 (210–651)	527 (184–812)	547 (497–597)	360 (222–634)	335 (204–660)	587 (220–692)
Person living with diabetes	196 (14)	42 (13)	65 (24)	25 (9)	17 (6)	47 (18)
Prior tuberculosis diagnosis	271 (19)	43 (13)	58 (21)	32 (12)	100 (38)	38 (15)
Symptoms						
Cough for ≥2 wk	1392 (100)	326 (100)	276 (100)	267 (100)	262 (100)	261 (100)
Fever	489 (35)	50 (15)	98 (36)	184 (69)	93 (35)	64 (25)
Hemoptysis	170 (12)	23 (7)	61 (22)	44 (16)	19 (7)	23 (9)
Night sweats	474 (34)	56 (17)	89 (32)	167 (63)	136 (52)	26 (10)
Weight loss	639 (46)	91 (28)	79 (29)	187 (70)	168 (64)	114 (44)
Loss of appetite	515 (37)	75 (23)	73 (26)	157 (59)	114 (44)	96 (37)
Reference standards						
Sputum Xpert Ultra						
Tuberculosis negative	1114 (80)	304 (93)	183 (66)	176 (66)	213 (81)	238 (91)
Tuberculosis positive	274 (20)	22 (7)	93 (34)	90 (34)	47 (18)	22 (8)
Indeterminate	4 (0)	0 (0)	0 (0)	1 (0)	2 (1)	1 (0)
Highest semiquantitative grade						
Trace	6 (2)	1 (5)	0 (0)	3 (3)	2 (4)	0 (0)
Very low	43 (16)	5 (23)	14 (15)	10 (11)	10 (20)	4 (18)
Low	85 (31)	8 (36)	37 (40)	19 (21)	12 (24)	9 (41)
Medium	68 (25)	6 (27)	21 (23)	23 (25)	12 (24)	6 (27)
High	75 (27)	2 (9)	21 (23)	36 (40)	13 (27)	3 (14)
MRS						
Tuberculosis negative	1089 (78)	297 (91)	171 (62)	174 (65)	209 (80)	238 (91)
Tuberculosis positive	303 (22)	29 (9)	105 (38)	93 (35)	53 (20)	23 (9)
Blood collection method						
Venous	1143 (82)	326 (100)	276 (100)	267 (100)	13 (5)	261 (100)
Capillary	249 (18)	0 (0)	0 (0)	0 (0)	249 (95)	0 (0)
Chest radiography type						
Digital	1148 (82)	326 (100)	276 (100)	23 (9)	262 (100)	261 (100)
Analogue	244 (18)	0 (0)	0 (0)	244 (91)	0 (0)	0 (0)

Abbreviations: BMI, body mass index; IQR, interquartile range; MRS, microbiologic reference standard; PWH, people with human immunodeficiency virus (HIV); SD, standard deviation.

^a^Data represent no. (%) of participants unless otherwise specified.

^b^BMI calculated as weight in kilograms divided by height in meters squared.

^c^HIV status was missing for 6 participants in South Africa.

### Diagnostic Accuracy

Xpert HR was conducted using venous (82% [n = 1143]) or capillary blood (18% [n = 249]). Chest radiographic images were collected digitally for 1148 (83%), and analogue images were scanned for 244 (18%). The distribution of each novel test quantitative result by MRS category is presented in the Supplementary Materials (Supplementary Table 2). The tuberculosis score output by Xpert HR was significantly lower in people with tuberculosis than in those without (*P* < .001), whereas the CRP result and CAD4TB score were both higher in people with tuberculosis (*P* < .001). There were no adverse events from any testing.

**Figure 2.**
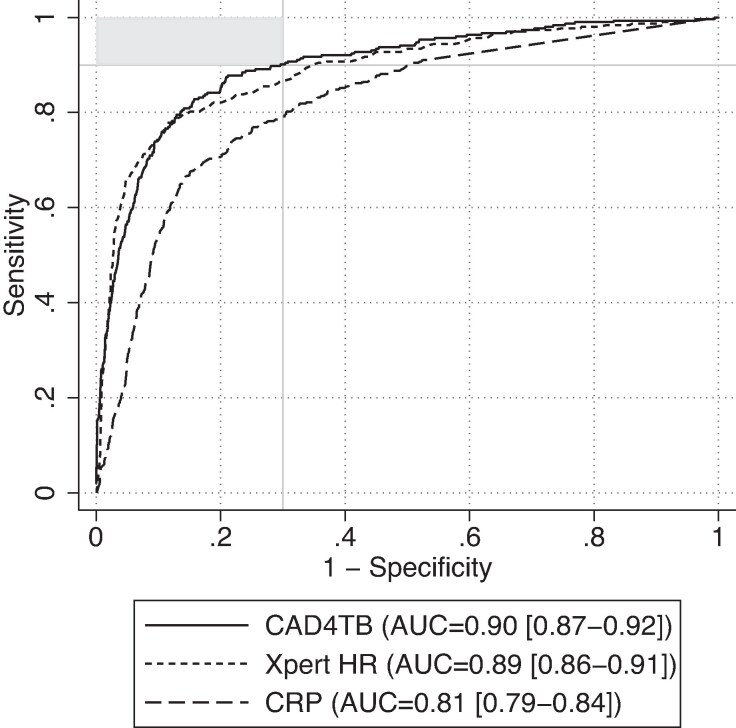
Receiver operating characteristic (ROC) curve (reference: microbiologic reference standard [MRS]). ROC curves against the MRS with area under the curve (AUC) and 95% confidence interval displayed for CAD4TB, Cepheid Xpert TB Host Response (Xpert HR), and C-reactive protein (CRP). Upper-left area shaded in gray notes the region where tests meet target product profile targets (≥90% sensitivity, ≥70% specificity). Of 1392 participants from the Philippines, Vietnam, Uganda, South Africa, and India with presumptive tuberculosis, 303 (22%) had microbiologically confirmed tuberculosis.

The AUC for CAD4TB was 0.90 (95% CI, .87–.92). Xpert HR had a similar AUC, 0.89 (95% CI, .86–.91), and CRP reached only 0.81 (.79–.84) (Figure 2). ROC analysis against the sputum Xpert reference standard is available in the Supplementary Materials (Supplementary Figure 2).

Against the MRS and at a sensitivity of 90%, CAD4TB (tuberculosis score, ≥28.61) achieved the highest specificity, 70.3% (95% CI, 67.5%–73.0%), and Xpert HR (tuberculosis score ≤−1.3) achieved a specificity of 65.1% (62.2%– 67.9%). CRP, with a cutoff of ≥2.81 mg/L, achieved a specificity of 49.7% (95% CI, 46.7%–52.7%). At the WHO-recommended cutoff of ≥5 mg/L, the sensitivity of CRP was 82.8% (95% CI, 78.1%–86.9%), and the specificity, 64.8% (61.9%–67.7%).

Evaluating CAD4TB, Xpert HR, and CRP in comparison using these cutoffs, CAD4TB performs best, with a specificity 5.2% (95% CI, 1.6%–8.9%) greater than that of Xpert HR, and 20.6% (17.0%–24.3%) greater than that of CRP (Table 2). Accuracy against the sputum Xpert Ultra alone is presented in the Supplementary Materials (Supplementary Table 6). We noted heterogeneity in diagnostic accuracy between countries and so did a sensitivity analysis stratified by country (Supplementary Table 7) but found similar results for sensitivity and specificity.

**Table 2.** Head-to-Head Comparison of Diagnostic Accuracy at Primary Cutoff Points

Screening Method	Quantitative Value (Cutoff) Indicating Positive Result^a^	Specificity (95% CI), %	Absolute Difference in Specificity (95% CI), %		
			vs CAD4TB	vs Xpert HR	vs CRP
1-Step screening					
CAD4TB^b^	Tuberculosis score ≥28.61	70.3 (67.5–73.0)	…	5.2 (1.6–8.9)	20.6 (17.0–24.3)
Xpert HR	Tuberculosis score ≤−1.3	65.1 (62.2–67.9)	−5.2 (−8.9 to −1.6)	…	15.4 (11.9–19.0)
CRP	≥2.81 mg/L	49.7 (46.7–52.7)	−20.6 (−24.3 to −17.0)	−15.4 (−19.0 to −11.9)	…
2-Step screening using sequential negative serial screening					
Xpert HR– CAD4TB^b^	CAD ≥49.68 or Xpert HR ≤−2.07	79.6 (77.1–82.0)	9.3 (6.6–11.9)	14.5 (11.4–17.6)	29.9 (26.5–33.4)
CRP-CAD4TB^b^	CRP ≥51.38 mg/L or CAD ≥37.77	75.9 (73.3–78.5)	5.6 (3.7–7.5)	10.8 (7.4–14.3)	26.3 (22.8–29.7)
CRP–Xpert HR	CRP ≥66.66 mg/L or Xpert HR ≤−1.34	64.2 (61.3–67.0)	−6.2 (−9.7 to −2.6)	−0.9 (−2.0 to −0.2)	14.5 (11.1–17.9)
2-Step screening using sequential positive serial screening^c^					
Xpert HR–CAD4TB^b^	Xpert HR ≤−0.81 and CAD ≥24.87	73.7 (71.0–76.3)	3.4 (1.5–5.3)	8.6 (5.4–11.9)	24.1 (20.6–27.5)
CRP-CAD4TB	CRP has no added value	…	…	…	…
CRP–Xpert HR	CRP has no added value	…	…	…	…

Abbreviations: CAD, computer-aided detection, represented by CAD4TB; CI, confidence interval; CRP, C-reactive protein; Xpert HR, Cepheid Xpert TB Host Response.

^a^Cutoff points were chosen to achieve ≥90% sensitivity and maximize specificity against the microbiologic reference standard in the overall study population.

^b^Meets current target product profile target (≥90% sensitivity, ≥70% specificity).

^c^The order of tests in 2-step screening does not affect the accuracy.

### Two-Test Screening Algorithms

**Figure 3.**
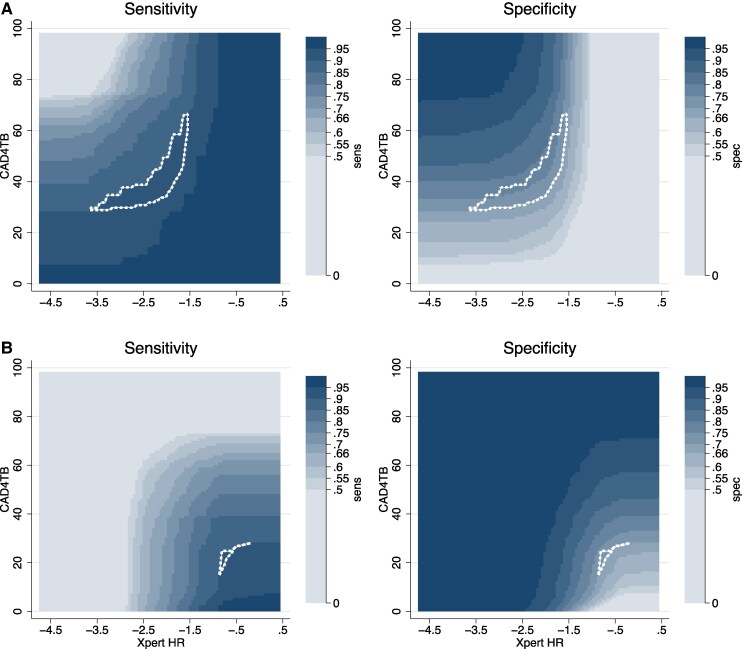
Selection of cutoff points for 2-step screening algorithm combining Cepheid Xpert TB Host Response (Xpert HR) and CAD4TB. *A,* Possible cutoffs using sequential negative serial screening, in which the second screening test is conducted only if results of the first are negative, and a positive screen is defined as positive on either test. *B,* Possible cutoffs using sequential positive serial screening, in which the second screening test is conducted only if results of the first are positive, and a positive screen is defined as positive on both tests. The x-axis shows all potential cutoff points for Xpert HR (test positive defined as less than or equal to the cutoff point chosen), and the y-axis shows all potential cutoff points for CAD4TB (test positive defined as greater than or equal to the cutoff chosen). Each point on the graph corresponds to a pair of cutoffs used to define a positive screening algorithm. For example, in *A,* a sequential negative test with test positive defined as CAD4TB score ≥40 or Xpert HR score≤−2 would have 91.7% sensitivity and 73.1% specificity. Shades represent the range of sensitivities and specificities possible, and the outlined region contains pairs with sensitivity ≥90% and specificity ≥70% (n = 417 in *A* and n = 31 in *B*).

We found that 417 pairs of cutoff points for a combined Xpert HR–CAD4TB test achieved TPP targets with a sequential negative serial screening approach, and 31 pairs of cutoff points achieved TPP targets with a sequential positive serial screening approach (Figure 3). The selection process for other 2-test screening algorithms is presented in the Supplementary Materials (Supplementary Figures 3 and 4). The optimal combination of CAD4TB and Xpert HR (cutoff, ≥49.68 for CAD4TB score and ≤−2.07 for Xpert HR score) achieved a specificity of 79.6% (95% CI, 77.1%–82.0%), with a sensitivity of 90%. This represents 9.3% (95% CI, 6.6%–11.9%) improvement over CAD4TB alone, 14.5% improvement over Xpert HR alone (11.4%–17.6%), and 29.9% improvement over CRP (26.5%–33.4%; Table 2). Other 2-step screening algorithms had lower accuracy. Using sequential negative serial screening, both Xpert HR–CAD4TB and CRP-CAD4TB met current TPP targets. Using sequential positive serial screening, only Xpert HR–CAD4TB met the current TPP targets (Table 2). The agreement between tests and test algorithms is reported in the Supplementary Materials (Supplementary Table 8).

### Subgroup Analyses

With use of the same cutoff points for the best-performing 2-test screening algorithm (Xpert HR–CAD4TB using sequential negative serial screening), accuracy varied most notably by country, with sensitivity ranging from 62.1% (95% CI, 42.3%–79.3%) in the Philippines to 100% (85.2%–100%) in India. Sensitivity was similar among people with and those without HIV (89.5% vs 90.2%, respectively) and among people with and those without diabetes (sensitivity, 91.7% vs 89.7%); specificity was similar among people with and those without diabetes (77.2% vs 80.0%, respectively) and slightly higher among people without HIV (81.1% vs 71.1% among those without HIV; Supplementary Figure 5). Female participants had lower sensitivity (81.4% vs 94.2% for male participants) but higher specificity (85.4% vs 74.0%, respectively; Supplementary Figure 5). Subgroup analyses of other combined tests are presented in the Supplementary Materials (Supplementary Figures 6–8).

Using subgroup-specific cutoff points, all 1- and 2-step screening algorithms except CRP alone achieved 90% sensitivity among participants in the Philippines (29 of 326 with MRS-positive tuberculosis) and among females (95 of 623 with MRS-positive tuberculosis). No screening algorithms met TPP targets among female participants, and only one screening algorithm, defined by Xpert HR score ≤−0.658 and CAD4TB score ≥6.727, met TPP targets among participants in the Philippines, with a specificity ≥70% (Supplementary Table 3).

In a hypothetical cohort of 1000 people with presumed tuberculosis, assuming a tuberculosis prevalence of 10%, the best-performing combined test would achieve a positive predictive value of 32.8% (95% CI, 27.3%–38.8%) and a negative predictive value of 98.6% (97.5%–99.3%) (Supplementary Table 4). A 10% prevalence is representative of peripheral settings for screening. Performing CAD4TB–Xpert HR using sequential negative serial screening (positive defined as CAD4TB score ≥49.68 or Xpert HR score ≤−2.07), followed by sputum testing if positive, would avert 726 sputum tests versus 643 averted with use of CAD4TB alone (Supplementary Table 5). Both screening strategies would miss 10 people (10%) with tuberculosis. However, to achieve this accuracy, performing CAD4TB–Xpert HR using sequential negative serial screening would require 73%–77% of participants to receive a second screening test, depending on order (Supplementary Table 5).

## DISCUSSION

Comparing CAD4TB, Xpert HR, and CRP, we found that CAD4TB was the most accurate tuberculosis triage test for pulmonary tuberculosis and the only test that met WHO TPP specificity targets (≥70% at ≥90% sensitivity) in a multicountry cohort of people with presumptive tuberculosis. Combining CAD4TB with Xpert HR or CRP using a sequential negative serial screening algorithm further improved accuracy, while only a combination with Xpert HR improved performance when using sequential positive serial screening. The diagnostic accuracy of CAD4TB in combination with other screening tests has not been well studied. Per-screen costs of certain CAD solutions, when used at scale, have proved to be less costly compared with a radiologist [22] and more effective in terms of timeliness and completeness of results when implemented among people with HIV [23].

In a hypothetical cohort of 1000 people with 10% tuberculosis prevalence, adding Xpert HR to CAD4TB using sequential negative serial screening averted 83 Xpert Ultra tests compared with CAD4TB alone (274 vs 357 requiring sputum Xpert testing); however, about three-quarters would require 2 screening tests before sputum testing. A sequential positive serial screening algorithm using the same tests would require fewer screening tests but more confirmatory testing (327 requiring sputum Xpert testing). Cost, however, is likely to be a major limitation of the implementation of a CAD4TB–Xpert HR screening approach, given that recent data showed that the Xpert HR cartridge is unlikely to achieve cost-effectiveness if implemented alone [24]. While CRP-CAD4TB has lower specificity (3.7% less than Xpert HR–CAD4TB), it would avert more radiographs and sputum Xpert tests and thus warrants consideration from an economic standpoint. An analysis of CRP-CAD4TB screening in Uganda suggested that it could be cost-effective [25].

Beyond cost, uptake of this intervention may be limited by access to the equipment needed (radiographic systems, GeneXpert instrument), especially at lower-level health facilities. The time it takes to complete screening using both screening tests may also reduce the effectiveness of this algorithm. This 2-step approach is currently considered by WHO in the revision of the TPP for tuberculosis screening. However, the added steps may further delay diagnosis or result in loss to follow-up, and this needs to be further studied with implementation [25].

We found that performance varied by country and sex could be mitigated by using subgroup-specific cutoffs for test positivity; however, this is likely impractical. Different cutoffs might increase the complexity of validation for policy purposes and implementation. At the same time, tailoring guidelines for use will be important, as using universal cutoffs may miss key subgroups.

Strengths of this study include its population: a large, well-characterized, diverse cohort of adults with presumptive tuberculosis across 5 countries. Prospective and concurrent evaluation of multiple tuberculosis triage tests allowed for a head-to-head comparison of these tests. This study is methodologically robust, with consistent implementation across sites and a thorough MRS. However, several limitations should be considered in the interpretation of the following findings. First, the prevalence of tuberculosis differed across study sites, ranging from 9% to 38%, which may also suggest differing states of tuberculosis disease at the time of presentation, which may explain some heterogeneity across sites. Second, there is limited generalizability beyond pulmonary tuberculosis; these results apply only to symptomatic pulmonary tuberculosis, and performance may differ in people unable to provide sputum and those with extrapulmonary tuberculosis. Finally, since culture testing was not conducted for participants with a positive Xpert Ultra result, we may overestimate tuberculosis prevalence if any Xpert results were positive due to prior tuberculosis; however, this should be uncommon and would have a small impact on our results.

In conclusion, current versions of CAD4TB achieve TPP targets for a tuberculosis triage test and outperform Xpert HR and CRP. Combining screening tests in algorithms further increases accuracy, but cost and implementation feasibility of these screening approaches will need to be evaluated.

## Supplementary Material

ciae549_Supplementary_Data

## Supplementary Data

Supplementary materials are available at *Clinical Infectious Diseases* online. Consisting of data provided by the authors to benefit the reader, the posted materials are not copyedited and are the sole responsibility of the authors, so questions or comments should be addressed to the corresponding author.

## Notes

***Author Contributions.*** P. N., A. C., and C. M. D. conceptualized the study. R. C., P. P. J. P., A. C., A. G. W., and C. M. D. designed the model framework. B. T., A. A., D. J. C., V. D., D. V. N., N. V. N., G. T., W. W., C. Y., and W.N. collected the data. R. C. curated the data. R. C., P. P. J. P., and A. G. W. conducted formal analysis. R. C. and P. P. J. P. visualized the data. R. C., B. T., A. A., A. G. W., C. M. D., and W.N. drafted the manuscript. All authors edited and approved the manuscript.

***Disclaimer.*** Study funders and product developers had no role in study design, data collection, data analysis, data interpretation, or writing of the manuscript.

***Data availability.*** Data are available at https://doi.org/10.11588/data/KGVQ4T.

***Financial support.*** This work was supported by the US National Institute of Allergy and Infectious Diseases (grant U01AI152087). Point-of-care CRP test kits and ichroma II machines were donated by Boditech Med, and Xpert HR and Xpert Ultra test kits were donated by Cepheid. Computer-aided detection was performed by the Foundation for Innovative New Diagnostics (FIND) at no cost to the study. The installation and use of the CAD4TB software evaluated in this study was provided free of charge by Delft Imaging to FIND.
