# Supplementary material for: Diagnostic Accuracy of Tuberculosis Screening Tests in a Prospective Multinational Cohort: Chest Radiography With Computer-Aided Detection, Xpert Tuberculosis Host Response, and C-Reactive Protein
Source: Clin Infect Dis. 2024 Nov 7;82(2):e239–47. doi: 10.1093/cid/ciae549 (PMC13016676; doi:10.1093/cid/ciae549)
Supplement: ciae549_Supplementary_Data [file ciae549_supplementary_data.docx]

**APPENDIX**

[Additional contributors from the R2D2 TB Network 2](#_Toc184974829)

[Table S1. R2D2 TB Network study enrolment sites and ethics committees 7](#_Toc184974830)

[Figure S1. Sequential testing algorithms. 8](#_Toc184974831)

[Table S2. Summary of index test results by reference standard classification 8](#_Toc184974832)

[Table S3. Results using subgroup-specific cut-points 9](#_Toc184974833)

[Table S4. Positive predictive value and negative predictive value in a hypothetical cohort of 1,000 with 10% TB prevalence 10](#_Toc184974834)

[Table S5. The potential number of sputum tests averted by using a triage test in a hypothetical cohort of 1000 with 10% TB prevalence 11](#_Toc184974835)

[Figure S2. Receiver operating characteristic curve (reference: sputum Xpert reference standard). 12](#_Toc184974836)

[Figure S3. Selection of cut points for two-step screening algorithm combining CRP and CAD4TB. 13](#_Toc184974837)

[Table S6. Head-to-head comparison of diagnostic accuracy against sputum Xpert reference standard. 15](#_Toc184974838)

[Table S7. Sensitivity and specificity against the microbiologic reference standard, calculated using inverse variance weighted random effects models, stratified by country. 16](#_Toc184974839)

[Figure S5. Subgroup analysis of two-step screening algorithm Xpert HR-CAD4TB using a sequential negative serial screening algorithm. 17](#_Toc184974840)

[Figure S6. Subgroup analysis of two-step screening algorithm CRP-CAD4TB using a sequential negative serial screening algorithm. 18](#_Toc184974841)

[Figure S7. Subgroup analysis of two-step screening algorithm CRP-Xpert HR using a sequential negative serial screening algorithm. 19](#_Toc184974842)

[Figure S8. Subgroup analysis of two-step screening algorithm Xpert HR-CAD4TB using a sequential negative serial screening algorithm. 20](#_Toc184974843)

[Table S8. Agreement between binary triage tests (percent agreement, Cohen’s Kappa statistic) 21](#_Toc184974844)

## Additional contributors from the R2D2 TB Network

| **First name** | **Surname** | **Affiliation** |
| --- | --- | --- |
| **India** |  |  |
| Shanmugasundaram | Elango | Christian Medical College, Vellore, India |
| Jerusha | Emmanuel | Christian Medical College, Vellore, India |
| Vinita | Ernest | Christian Medical College, Vellore, India |
| Priyadarshini | Gajendran | Christian Medical College, Vellore, India |
| Flavita | John | Christian Medical College, Vellore, India |
| Bharath | Karthikeyan | Christian Medical College, Vellore, India |
| Divya | Mangal | Christian Medical College, Vellore, India |
| Swetha | Sankar | Christian Medical College, Vellore, India |
| Rajasekar | Sekar | Christian Medical College, Vellore, India |
| Reena | Sekar | Christian Medical College, Vellore, India |
| Deepa | Shankar | Christian Medical College, Vellore, India |
| Mary | Shibiya | Christian Medical College, Vellore, India |
| Sai | Vijayasree | Christian Medical College, Vellore, India |
| **Philippines** |  |  |
| Jared | Almonte | De La Salle Medical and Health Sciences Institute, Cavite |
| Kevin Joshua | Alonzo | National TB Reference Laboratory, Research Institute for Tropical Medicine, Department of Health |
| Mary Faith | Angcaya | De La Salle Medical and Health Sciences Institute, Cavite |
| Joseph Edwin L. | Bascuña | National TB Reference Laboratory, Research Institute for Tropical Medicine, Department of Health |
| Ramon P. | Basilio | National TB Reference Laboratory, Research Institute for Tropical Medicine, Department of Health |
| Asella Ruvijean | Cariaga | De La Salle Medical and Health Sciences Institute, Cavite, Philippines |
| Gabriella | Castillon | De La Salle Medical and Health Sciences Institute, Cavite |
| Karlo | Dayawon | De La Salle Medical and Health Sciences Institute, Cavite |
| Raul | Destura | National Institutes of Health, University of the Philippines Manila |
| Jezreel | Esguerra | De La Salle Medical and Health Sciences Institute, Cavite |
| Eleonor | Garcia | De La Salle Medical and Health Sciences Institute, Cavite |
| Darecil | Gelina | De La Salle Medical and Health Sciences Institute, Cavite |
| Joseph Aldwin | Goleña | De La Salle Medical and Health Sciences Institute, Cavite |
| Maria Marissa | Golla | De La Salle Medical and Health Sciences Institute, Cavite |
| Emmanuelle | Gutierrez | De La Salle Medical and Health Sciences Institute, Cavite |
| Gidalthi Jonathan | Ilagan | De La Salle Medical and Health Sciences Institute, Cavite |
| Dodge R. | Lim | National TB Reference Laboratory, Research Institute for Tropical Medicine, Department of Health |
| Jaiem | Maranan | De La Salle Medical and Health Sciences Institute, Cavite |
| Danaida | Marcelo | De La Salle Medical and Health Sciences Institute, Cavite |
| Leonedy | Masangcay | De La Salle Medical and Health Sciences Institute, Cavite |
| Jenkin | Mendoza | National TB Reference Laboratory, Research Institute for Tropical Medicine, Department of Health |
| Angelita | Pabruada | De La Salle Medical and Health Sciences Institute, Cavite |
| Laarean | Perlas | De La Salle Medical and Health Sciences Institute, Cavite |
| Annalyn | Reyes | De La Salle Medical and Health Sciences Institute, Cavite |
| Roeus Vincent Arjay G. | Reyes | National TB Reference Laboratory, Research Institute for Tropical Medicine, Department of Health |
| Lorenzo | Reyes | National TB Reference Laboratory, Research Institute for Tropical Medicine, Department of Health |
| Maria Guileane | Sanchez-Pogosa | National TB Reference Laboratory, Research Institute for Tropical Medicine, Department of Health |
| Maricef | Tonquin | De La Salle Medical and Health Sciences Institute, Cavite |
| **South Africa** |  |  |
| Shima | Abdulgadar | DSI-NRF Centre of Excellence for Biomedical Tuberculosis Research, South African Medical Research Council Centre for Tuberculosis Research, Division of Molecular Biology and Human Genetics, Faculty of Medicine and Health Sciences, Stellenbosch University, Cape Town, South Africa |
| Cammy | Botha | DSI-NRF Centre of Excellence for Biomedical Tuberculosis Research, South African Medical Research Council Centre for Tuberculosis Research, Division of Molecular Biology and Human Genetics, Faculty of Medicine and Health Sciences, Stellenbosch University, Cape Town, South Africa |
| Brigitta | Derendinger | DSI-NRF Centre of Excellence for Biomedical Tuberculosis Research, South African Medical Research Council Centre for Tuberculosis Research, Division of Molecular Biology and Human Genetics, Faculty of Medicine and Health Sciences, Stellenbosch University, Cape Town, South Africa |
| Jane | Fortuin | DSI-NRF Centre of Excellence for Biomedical Tuberculosis Research, South African Medical Research Council Centre for Tuberculosis Research, Division of Molecular Biology and Human Genetics, Faculty of Medicine and Health Sciences, Stellenbosch University, Cape Town, South Africa |
| Siphosethu | Gonya | DSI-NRF Centre of Excellence for Biomedical Tuberculosis Research, South African Medical Research Council Centre for Tuberculosis Research, Division of Molecular Biology and Human Genetics, Faculty of Medicine and Health Sciences, Stellenbosch University, Cape Town, South Africa |
| Chumani | Hatile | DSI-NRF Centre of Excellence for Biomedical Tuberculosis Research, South African Medical Research Council Centre for Tuberculosis Research, Division of Molecular Biology and Human Genetics, Faculty of Medicine and Health Sciences, Stellenbosch University, Cape Town, South Africa |
| Megan | Hendrikse | DSI-NRF Centre of Excellence for Biomedical Tuberculosis Research, South African Medical Research Council Centre for Tuberculosis Research, Division of Molecular Biology and Human Genetics, Faculty of Medicine and Health Sciences, Stellenbosch University, Cape Town, South Africa |
| Charlotte | Lawn | DSI-NRF Centre of Excellence for Biomedical Tuberculosis Research, South African Medical Research Council Centre for Tuberculosis Research, Division of Molecular Biology and Human Genetics, Faculty of Medicine and Health Sciences, Stellenbosch University, Cape Town, South Africa |
| Disha | Mathoorah | DSI-NRF Centre of Excellence for Biomedical Tuberculosis Research, South African Medical Research Council Centre for Tuberculosis Research, Division of Molecular Biology and Human Genetics, Faculty of Medicine and Health Sciences, Stellenbosch University, Cape Town, South Africa |
| Desiree Lem | Mbu | DSI-NRF Centre of Excellence for Biomedical Tuberculosis Research, South African Medical Research Council Centre for Tuberculosis Research, Division of Molecular Biology and Human Genetics, Faculty of Medicine and Health Sciences, Stellenbosch University, Cape Town, South Africa |
| Zintle | Ntetha | DSI-NRF Centre of Excellence for Biomedical Tuberculosis Research, South African Medical Research Council Centre for Tuberculosis Research, Division of Molecular Biology and Human Genetics, Faculty of Medicine and Health Sciences, Stellenbosch University, Cape Town, South Africa |
| Anna | Okunola | DSI-NRF Centre of Excellence for Biomedical Tuberculosis Research, South African Medical Research Council Centre for Tuberculosis Research, Division of Molecular Biology and Human Genetics, Faculty of Medicine and Health Sciences, Stellenbosch University, Cape Town, South Africa |
| Zaida | Palmer | DSI-NRF Centre of Excellence for Biomedical Tuberculosis Research, South African Medical Research Council Centre for Tuberculosis Research, Division of Molecular Biology and Human Genetics, Faculty of Medicine and Health Sciences, Stellenbosch University, Cape Town, South Africa |
| Fikiswa | Seti | DSI-NRF Centre of Excellence for Biomedical Tuberculosis Research, South African Medical Research Council Centre for Tuberculosis Research, Division of Molecular Biology and Human Genetics, Faculty of Medicine and Health Sciences, Stellenbosch University, Cape Town, South Africa |
| Charmaine | Van Der Walt | DSI-NRF Centre of Excellence for Biomedical Tuberculosis Research, South African Medical Research Council Centre for Tuberculosis Research, Division of Molecular Biology and Human Genetics, Faculty of Medicine and Health Sciences, Stellenbosch University, Cape Town, South Africa |
| Lusanda | Yekani | DSI-NRF Centre of Excellence for Biomedical Tuberculosis Research, South African Medical Research Council Centre for Tuberculosis Research, Division of Molecular Biology and Human Genetics, Faculty of Medicine and Health Sciences, Stellenbosch University, Cape Town, South Africa |
| **Uganda** |  |  |
| Lucy | Asege | Walimu, Kampala, Uganda |
| Alice | Bukirwa | Walimu, Kampala, Uganda |
| David | Katumba | Walimu, Kampala, Uganda |
| Esther | Kisakye | Walimu, Kampala, Uganda |
| Wilson | Mangeni | Walimu, Kampala, Uganda |
| Job | Mukwatamundu | Walimu, Kampala, Uganda |
| Sandra | Mwebe | Walimu, Kampala, Uganda |
| Annet | Nakaweesa | Walimu, Kampala, Uganda |
| Martha | Nakaye | Walimu, Kampala, Uganda |
| Talemwa | Nalugwa | Walimu, Kampala, Uganda |
| Irene | Nassuna | Walimu, Kampala, Uganda |
| Irene | Nekesa | Walimu, Kampala, Uganda |
| Justine | Nyawere | Walimu, Kampala, Uganda |
| John Baptist | Ssonko | Walimu, Kampala, Uganda |
| **Vietnam** |  |  |
| Hai | Dang | Vietnam National Tuberculosis Program-University of California San Francisco Research Collaboration Unit; Center for Promotion of Advancement of Society, Hanoi, Vietnam |
| Luong | Dinh | Vietnam National Lung Hospital |
| Hang | Do | Hanoi Lung Hospital, Hanoi, Vietnam |
| Tam | Do | Hanoi Lung Hospital, Hanoi, Vietnam |
| Thuong | Do | Vietnam National Lung Hospital |
| Dung | Dao | Hanoi Lung Hospital, Hanoi, Vietnam |
| Ha | Doan | National TB reference Lab/ Vietnam National Lung Hospital, Hanoi, Vietnam |
| Thien | Doan | Hanoi Lung Hospital, Hanoi, Vietnam |
| Huy | Ha | Vietnam National Tuberculosis Program-University of California San Francisco Research Collaboration Unit, Center for Promotion of Advancement of Society, Hanoi, Vietnam |
| Oanh | Lai | Hanoi Lung Hospital, Hanoi, Vietnam |
| Hien | Le | Vietnam National Tuberculosis Program-University of California San Francisco Research Collaboration Unit; Center for Promotion of Advancement of Society, Hanoi, Vietnam |
| Nguyet | Le | National TB reference Lab/ Vietnam National Lung Hospital, Hanoi, Vietnam |
| Anh | Nguyen | Hanoi Lung Hospital, Hanoi, Vietnam |
| Hanh | Nguyen | Vietnam National Tuberculosis Program-University of California San Francisco Research Collaboration Unit; Center for Promotion of Advancement of Society, Hanoi, Vietnam |
| Hoa | Nguyen | Vietnam National Lung Hospital |
| Hoang | Nguyen | Hanoi Lung Hospital, Hanoi, Vietnam |
| Thanh | Nguyen | Hanoi Lung Hospital, Hanoi, Vietnam |
| Yen | Nguyen | Hanoi Lung Hospital, Hanoi, Vietnam |
| Ha | Phan | Vietnam National Tuberculosis Program-University of California San Francisco Research Collaboration Unit, Center for Promotion of Advancement of Society, Hanoi, Vietnam |
| Nam | Pham | Vietnam National Tuberculosis Program-University of California San Francisco Research Collaboration Unit, Hanoi Lung Hospital, Hanoi, Vietnam |
| Thuong | Pham | Hanoi Lung Hospital, Hanoi, Vietnam |
| Trang | Trinh | Vietnam National Tuberculosis Program-University of California San Francisco Research Collaboration Unit, Center for Promotion of Advancement of Society, Hanoi, Vietnam |
| Phuong | Vu | Hanoi Lung Hospital, Hanoi, Vietnam |
| Trung | Vu | National TB reference Lab/ Vietnam National Lung Hospital, Hanoi, Vietnam |
| **USA** |  |  |
| Robert | Castro | University of California San Francisco, San Francisco, CA, USA |
| Adithya | Cattamanchi | University of California Irvine, Irvine, CA, USA |
| Catherine | Cook | University of California San Francisco, San Francisco, CA, USA |
| Sophie | Huddart | University of California San Francisco, San Francisco, CA, USA |
| Devan | Jaganath | University of California San Francisco, San Francisco, CA, USA |
| Midori | Kato-Maeda | University of California San Francisco, San Francisco, CA, USA |
| Tessa | Mochizuki | University of California San Francisco, San Francisco, CA, USA |
| Ruvandhi | Nathavitharana | Beth Israel Deaconess Medical Center, Harvard Medical School, Boston, MA, USA |
| Payam | Nahid | University of California San Francisco, San Francisco, CA, USA |
| Kevin | Nolan | University of California San Francisco, San Francisco, CA, USA |
| Kinari | Shah | University of California San Francisco, San Francisco, CA, USA |
| Swati | Sudarsan | University of California San Francisco, San Francisco, CA, USA |
| Christina | Yoon | University of California San Francisco, San Francisco, CA, USA |
| **Germany** |  |  |
| Maria del Mar | Castro Noriega | Heidelberg University Hospital, Heidelberg, Germany |
| Theresa | Pfurtscheller | Heidelberg University Hospital, Heidelberg, Germany |
| Seda | Yerlikaya | Heidelberg University Hospital, Heidelberg, Germany |
| **Switzerland** | | |
| Matthew | Arentz | FIND, Geneva, Switzerland |
| Nathalie | Frey | FIND, Geneva, Switzerland |
| Sam | Linsen | FIND, Geneva, Switzerland |

## Table S1. R2D2 TB Network study enrolment sites and ethics committees

| City, Country | Enrollment sites | Ethics Committee* |
| --- | --- | --- |
| Vellore, India | CMC Pulmonary Outpatient Department, Primary care clinics in Vellore (Shalom/LCC, Chittor, CHAD) and Chitoor (CMC satellite campus) | Christian Medical College Institutional Review Board (13256) |
| Hanoi, Vietnam | Outpatient departments, Hanoi Lung Hospital | Ministry of Health Ethical Committee for National Biological Medical Research (94/CN-HĐĐĐ); National Lung Hospital Ethical Committee for Biological Medical Research (566/2020/NCKH); Hanoi Lung Hospital Science and Technology Initiative Committee (22/BVPHN) |
| Dasmariñas City, Philippines | Community-based screening in Dasmariñas City and nearby municipalities, outpatient clinics in Dasmariñas City | De La Salle Health Sciences Institute Independent Ethics Committee (2020-33-02-A) |
| Cape Town,  South Africa | Scottsdene and Wallacedene primary care clinics; Brooklyn Chest Hospital; Khayelitsha District Health Center; Kraaifontein Community Health Clinic | Stellenbosch University Health Research Ethics Committee (M20/07/020) |
| Kampala, Uganda | Mulago Outpatient Department, Kisenyi Health Center, | Makerere University, College of Health Sciences, School of Medicine, Research Ethics Committee (2020-182) |

* The study was additionally approved by the University of California San Francisco Institutional Review Board (20-32670), and the University of Heidelberg Ethics Committee of

the Medical Faculty (S-539/2020)

## Figure S1. Sequential testing algorithms.

For each potential combination of screening tests, we considered a sequential negative serial screening approach (panel A) and a sequential positive serial screening approach (panel B).

## Table S2. Summary of index test results by reference standard classification

| **Microbiologic Reference Standard** | | | | |
| --- | --- | --- | --- | --- |
| **Index test, mean (SD)** | **Positive (n=303)** | | **Negative (n=1,089)** | |
| CRP | 61.0 (65.3) | | 15.6 (37.4) | |
| Xpert HR | -2.67 (0.94) | | -1.15 (0.70) | |
| CAD4TB | 68.2 (26.0) | | 20.1 (23.2) | |
| **Sputum Xpert Reference Standard** | | | | |
| **Index test, mean (SD)** | **Positive (n=274)** | **Negative (n=1,114)** | | **Indeterminate (n=4)** |
| CRP | 65.3 (65.9) | 15.6 (37.5) | | 43.6 (36.2) |
| Xpert HR | -2.79 (0.88) | -1.16 (0.70) | | -2.19 (1.13) |
| CAD4TB | 69.6 (25.1) | 20.9 (24.0) | | 46.1 (39.2) |

SD: standard deviation

## Table S3. Results using subgroup-specific cut-points

|  | Using Philippines-specific cut-points  (N=326 people in the Philippines,  29 [9%] with TB) | | Using female-specific cut-points  (N=623 females,  95 [15.3%] with TB) | |
| --- | --- | --- | --- | --- |
|  | Quantitative value indicating positive test | Specificity  (95% CI) | Quantitative value indicating positive test | Specificity  (95% CI) |
| One-step screening | | | | |
| CAD4TB | TB score ≥7.1697 | 61.6%  (55.8, 67.2) | TB score≥6.005 | 53.7%  (49.4, 58.0) |
| Xpert HR | TB score ≤-0.8499 | 45.5%  (39.7, 51.3) | TB score≤1.15 | 54.1%  (49.8, 58.4) |
| CRP | Did not achieve 90% sensitivity |  | Did not achieve 90% sensitivity |  |
| Two-step screening using either test positive approach | | | | |
| Xpert HR-CAD4TB | Xpert HR ≤-2.69 or  CAD4TB score ≥6.73 | 60.3%  (54.5, 65.9) | Xpert HR ≤-1.318 or  CAD4TB score ≥62.393 | 63.8%  (59.6, 67.9) |
| CRP-CAD4TB | CRP≥10.79 or  CAD4TB score≥10.49 | 65.0%  (59.3, 70.4) | CRP≥36.105 or  CAD4TB score≥10.988 | 61.9%  (57.7, 66.1) |
| CRP-Xpert HR | CRP≥23.22 or  Xpert HR≤-0.81 | 45.5%  (39.7, 51.3) | CRP≥23.224 or  Xpert HR≤-1.116 | 53.7%  (49.4, 58.0) |
| Two-step screening using both test positive approach | | | | |
| Xpert HR-CAD4TB* | Xpert HR≤-0.658 and  CAD4TB≥6.727 | 71.4%  (65.9, 76.5) | Xpert HR≤-1.015 and  CAD4TB≥3.079 | 63.2%  (59.0, 67.3) |
| CRP-CAD4TB | CRP has no added value | - | - | - |
| CRP-Xpert HR | CRP has no added value | - | - | - |

CI: confidence interval; CAD: computer aided detection; CRP: C-reactive protein; HR: host response; TPP: target product profile

* Meets current TPP target in the Philippines (≥90% sensitivity, ≥70% specificity)

^ Meets current TPP target among females (≥90% sensitivity, ≥70% specificity)

## Table S4. Positive predictive value and negative predictive value in a hypothetical cohort of 1,000 with 10% TB prevalence

|  | **Positive predictive value**  **(95% CI)**  **n=100 people with TB** | **Negative predictive value**  **(95% CI)**  **(n=900 people without TB)** |  |
| --- | --- | --- | --- |
| **One-step screening** | | | |
| CAD4TB | 25.2% (20.8, 30.0) | 98.4% (97.2, 99.3) |  |
| Xpert HR | 22.3% (18.3, 26.7) | 98.3% (96.9, 99.2) |  |
| CRP | 16.6% (13.5, 20.0) | 97.8% (96.0, 98.9) |  |
| **Two-step screening using either test positive approach** | | | |
| Xpert HR​-CAD4TB* | 32.8% (27.3, 38.8) | 98.6% (97.5, 99.3) |  |
| CRP​-CAD4TB* | 29.3% (24.3, 34.8) | 98.6% (97.4, 99.3) |  |
| CRP-Xpert HR**​** | 21.8% (17.9, 26.2) | 98.3% (96.9, 99.2) |  |
| **Two-step screening using both test positive approach** | | | |
| Xpert HR-CAD4TB* | 27.5% (22.8, 32.7) | 98.5% (97.3, 99.3) |  |

CI: confidence interval

## Table S5. The potential number of sputum tests averted by using a triage test in a hypothetical cohort of 1000 with 10% TB prevalence

|  | **Number that have first test performed** (total population) | **Number that have second test performed** | **Number of people with TB missed**  (n=100 with TB) | **Number of people correctly classified by triage test** | **Number of people triage test positive, requiring sputum testing**  (N=1000 with presumptive TB) | **Number of sputum tests averted**  (N=1000 with presumptive TB) |  |
| --- | --- | --- | --- | --- | --- | --- | --- |
| **One-step screening** | | | | | | | |
| CAD4TB | 1,000 | 0 | 10 (10%) | 723 (72.3%) | 357 (35.7%) | 643 |  |
| Xpert HR | 1,000 | 0 | 10 (10%) | 676 (67.6%) | 404 (40.4%) | 596 |  |
| CRP | 1,000 | 0 | 10 (10%) | 537 (53.7%) | 543 (54.3%) | 457 |  |
| **Two-step screening using either test positive approach** | | | | | | | |
| Xpert HR​-CAD4TB*  CAD4TB-Xpert HR* | 1,000 | 765  728 | 10 (10%) | 806 (80.6%) | 274 (27.4%) | 726 |  |
| CRP​-CAD4TB*  CAD4TB-CRP* | 1,000 | 851  643 | 10 (10%) | 773 (77.3%) | 307 (30.7%) | 693 |  |
| CRP-Xpert HR**​**  Xpert HR-CRP | 1,000 | 884  541 | 10 (10%) | 668 (66.8%) | 412 (41.2%) | 588 |  |
| **Two-step screening using both test positive approach** | | | | | | | |
| Xpert HR-CAD4TB*  CAD4TB-Xpert HR* | 1,000 | 712  463 | 10 (10%) | 753 (75.3%) | 327 (32.7%) | 673 |  |
| CAD4TB-CRP | 1,000 | 435 | 10 (10%) | 716 (71.6%) | 364 (36.4%) | 636 |  |
| Xpert HR-CRP | 1,000 | 476 | 10 (10%) | 670 (67%) | 410 (41%) | 590 |  |

Note. The order of tests in two-step screening does not impact the accuracy.

* Meets current TPP target (≥90% sensitivity, ≥70% specificity)

Figure S2. Receiver operating characteristic curve (reference: sputum Xpert reference standard). ROC curves against the sputum Xpert reference standard with AUC and 95% CI displayed for CAD4TB, Xpert HR, and CRP. The upper-left area shaded in gray notes the region where tests meet TPP targets (≥90% sensitivity, ≥70% specificity). N=1,388 participants from the Philippines, Vietnam, Uganda, South Africa, and India with presumptive TB (n=274, 20% with sputum Xpert-positive TB). 4 participants included in the primary analysis had an indeterminate sputum Xpert Ultra result and were excluded from this analysis.

Figure S3. Selection of cut points for two-step screening algorithm combining CRP and CAD4TB. Panel (A) shows the possible cut-points using the sequential negative serial screening approach, in which the second screening test is conducted only if the first is negative and a positive screen is defined as positive on either test. Panel (B) shows the possible cut-points using the sequential positive serial screening approach, in which the second screening test is conducted only if the first is positive and a positive screen is defined as positive on both tests. The x-axis shows all potential cut-points for CAD4TB (test positive defined as greater than or equal to the cut point chosen), and the y-axis shows all potential cut points for CRP (test positive defined as greater than or equal to the cut point chosen). Each point on the graph corresponds to a pair of cut points used to define a positive screening algorithm. The colors represent the range of sensitivities and specificities possible. The outlined region contains pairs with sensitivity≥90% and specificity ≥70% (n=310 in panel A, n=0 in panel B).

**Figure S4. Selection of cut points for two-step screening algorithm combining CRP and Xpert HR.** Panel (A) shows the possible cut-points using the sequential negative serial screening approach, in which the second screening test is conducted only if the first is negative and a positive screen is defined as positive on either test. Panel (B) shows the possible cut-points using the sequential positive serial screening approach, in which the second screening test is conducted only if the first is positive and a positive screen is defined as positive on both tests. The x-axis shows all potential cut-points for Xpert HR (test positive defined as less than or equal to the cut point chosen), and the y-axis shows all potential cut points for CRP (test positive defined as greater than or equal to the cut point chosen). Each point on the graph corresponds to a pair of cut points used to define a positive screening algorithm. The colors represent the range of sensitivities and specificities possible. No pair of cut points using either approach achieved sensitivity≥90% and specificity ≥70%.

Table S6. Head-to-head comparison of diagnostic accuracy against sputum Xpert reference standard. Cut points were chosen to achieve ≥90% sensitivity and maximize specificity against the sputum Xpert reference standard.

| N=1,388 | Quantitative value indicating  positive test | Specificity  (95% CI) | Absolute difference in specificity  (95% CI) | | | |  |
| --- | --- | --- | --- | --- | --- | --- | --- |
|  |  |  | **vs. CAD4TB** | **vs. Xpert HR** | | **vs. CRP** | |
| One-step screening | | | | | | |  |
| CAD4TB* | TB score ≥34.08 | 74.7%  (72.0, 77.2) | - | 1.7  (-1.7, 5.1) | 15.4  (11.9, 18.8) | |  |
| Xpert HR* | TB score ≤-1.499 | 73.0%  (70.3, 75.6) | -1.7  (-5.1, 1.7) | - | 13.6%  (10.3, 17.0) | |  |
| CRP | ≥4.07 mg/L | 59.3%  (56.4, 62.2) | -15.4  (-18.8, -11.9) | -13.6%  (-17.0, -10.3) | - | |  |
| Two-step screening using either test positive appraoch | | | | | | |  |
| Xpert HR-CAD4TB* | CAD4TB ≥62.59 or  Xpert HR ≤-2.28 | 85.1%  (82.9, 87.1) | 10.4  (7.9, 12.9) | 12.1  (9.4, 14.9) | 25.8  (22.6, 29.0) | | |
| CRP-CAD4TB* | CRP ≥45.71 mg/L or  CAD4TB ≥39.16 | 78.0%  (75.5, 80.4) | 3.3  (1.3, 5.3) | 5.0  (1.8, 8.3) | 18.7  (15.5, 21.8) | | |
| CRP-Xpert HR* | CRP ≥265.23mg/L or  Xpert HR ≤-1.495 | 72.9%  (70.2, 75.5) | -1.8  (-5.2, 1.6) | 0.1  (-0.4, 0.2) | 13.6  (10.2, 16.9) | | |
| Two-step screening using both test positive approach | | | | | | |  |
| Xpert HR-CAD4TB* | CAD4TB ≥31.82 and  Xpert HR ≤-0.76 | 77.6%  (75.1, 80.1) | 3.0  (1.6, 4.3) | 4.7  (1.5, 7.9) | 18.3  (15.0, 21.6) | | |
| CRP-CAD4TB | CRP has no added value | - | - | - | - | | |
| CRP-Xpert HR | CRP has no added value | - | - | - | - | | |

CI: confidence interval; CAD: computer aided detection, represented by CAD4TB; CRP: C-reactive protein; HR: host response; TPP: target product profile

N=1,392 people with presumptive TB, n=274 with sputum Xpert Ultra positive TB

Note. 4 participants included in the primary analysis had an indeterminate sputum Xpert Ultra result and were excluded from this analysis.

* Meets current TPP target (≥90% sensitivity, ≥70% specificity)

Table S7. Sensitivity and specificity against the microbiologic reference standard, calculated using inverse variance weighted random effects models, stratified by country. This sensitivity analysis was done to explore the impact of heterogeneity by country on the estimates of sensitivity and specificity. The same cut-points identified in the primary analysis were used here. Cut-points were chosen to achieve ≥90% sensitivity and maximize specificity against the microbiologic reference standard in the overall study population.

|  | Quantitative value indicating positive test | Sensitivity  (95% CI) | Specificity  (95% CI) |
| --- | --- | --- | --- |
| One-step screening | | | |
| CAD4TB | TB score ≥28.61 | 90.9%  (85.2, 96.6) | 68.7%  (58.1, 79.3) |
| Xpert HR | TB score ≤-1.3 | 91.0%  (87.2, 94.7) | 64.3%  (58.0, 70.7) |
| CRP | ≥2.81 mg/L | 91.1%  (85.4, 96.8) | 48.9%  34.0, 63.7) |
| Two-step screening using sequential negative serial screening | | | |
| Xpert HR- CAD4TB | CAD ≥49.68 or  Xpert HR ≤-2.07 | 89.4%* (81.9, 97.0) | 78.8%  (71.1, 86.6) |
| CRP- CAD4TB | CRP ≥51.38 mg/L  or CAD ≥37.77 | 90.9%  (85.3, 96.6) | 74.7%  (65.4, 84.1) |
| CRP-Xpert HR | CRP≥ 66.66 or  Xpert HR≤-1.34 | 91.1%  (87.6, 94.7) | 63.2%  (55.0, 71.3) |
| Two-step screening using sequential positive serial screening | | | |
| Xpert HR-CAD4TB* | Xpert HR≤-0.81  and CAD≥24.87 | 90.3%  (83.7, 97.0) | 72.2%  (61.9, 82.5) |
| CRP-CAD4TB | CRP has no added value | - | - |
| CRP-Xpert HR | CRP has no added value | - | - |

CI: confidence interval; CAD: computer aided detection, represented by CAD4TB; CRP: C-reactive protein; HR: host response; TPP: target product profile

Note. The order of tests in two-step screening does not impact the accuracy.

*India was dropped from the pooled estimate of sensitivity of this test, as the sensitivity in India was 100% with a standard error of 0.

## Figure S5. Subgroup analysis of two-step screening algorithm Xpert HR-CAD4TB using a sequential negative serial screening algorithm.

## Figure S6. Subgroup analysis of two-step screening algorithm CRP-CAD4TB using a sequential negative serial screening algorithm.

## Figure S7. Subgroup analysis of two-step screening algorithm CRP-Xpert HR using a sequential negative serial screening algorithm.

## Figure S8. Subgroup analysis of two-step screening algorithm Xpert HR-CAD4TB using a sequential negative serial screening algorithm.

## Table S8. Agreement between binary triage tests (percent agreement, Cohen’s Kappa statistic)

| **Percent agreement (Kappa)** | **CAD4TB** | **Xpert HR** | **CRP** |  | **CAD-Xpert HR (sequential negative)** | **CAD-CRP (sequential negative)** | **CRP-Xpert HR (sequential negative)** |  | **CAD-Xpert HR (sequential positive)** | **CAD-CRP (sequential positive)** | **CRP-Xpert HR (sequential positive)** |
| --- | --- | --- | --- | --- | --- | --- | --- | --- | --- | --- | --- |
| **CAD4TB** | - | 69.0%  (0.38) | 65.5%  (0.33) |  | 83.3%  (0.65) | 92.0%  (0.84) | 70.0%  (0.40) |  | 92.0%  (0.84) | 99.4%  (0.99) | 69.0%  (0.37) |
| **Xpert HR** | - | - | 69.4% (0.39) |  | 76.2%  (0.51) | 71.8% (0.43) | 97.4%  (0.95) |  | 75.1%  (0.50) | 69.4%  (0.38) | 99.4%  (0.99) |
| **CRP** | - | - | - |  | 66.1%  (0.36) | 66.8%  (0.36) | 71.1%  (0.43) |  | 67.0%  (0.36) | 65.8%  (0.33) | 69.8%  (0.40) |
|  |  |  |  |  |  |  |  |  |  |  |  |
| **CAD-Xpert HR (sequential negative)** | - | - | - |  | - | 88.5%  (0.75) | 77.0%  (0.53) |  | 83.5%  (0.65) | 82.9%  (0.64) | 75.7%  (0.51) |
| **CAD-CRP (sequential negative)** | - | - | - |  | - | - | 73.9%  (0.47) |  | 88.9%  (0.77) | 91.8%  (0.83) | 71.6%  (0.43) |
| **CRP-Xpert HR (sequential negative)** | - | - | - |  | - | - | - |  | 75.7%  (0.51) | 70.6%  (0.41) | 97.1%  (0.94) |
|  |  |  |  |  |  |  |  |  |  |  |  |
| **CAD-Xpert HR (sequential positive)** | - | - | - |  | - | - | - |  | - | 92.5%  (0.85) | 75.1%  (0.50) |
| **CAD-CRP (sequential positive)** | - | - | - |  | - | - | - |  | - | - | 69.3%  (0.38) |
| **CRP-Xpert HR (sequential positive)** | - | - | - |  | - | - | - |  | - | - | - |
